# P-729. Limit of Detection and Inclusivity of a Research-Use-Only (RUO) Prototype of a Multiplexed Sample-to-Answer Diagnostic System for Identification of Sexually Transmitted Infections from Vaginal Swabs and Penile Urine

**DOI:** 10.1093/ofid/ofaf695.940

**Published:** 2026-01-11

**Authors:** Kerrin Koch, Marta Mangifesta, Caitlin Castañeda, Lorin Brookes, Cameron Hill, Jeremiah Antosch, Sarah Gross, Ray Kirby Jennings, Jenifer Einstein, Tanner Robinson, Justin Keener, Usha Spaulding, Margarita Rogatcheva

**Affiliations:** bioMérieux, Salt Lake City, UT; bioMérieux, Salt Lake City, UT; bioMérieux, Salt Lake City, UT; bioMérieux, Salt Lake City, UT; bioMérieux, Salt Lake City, UT; bioMérieux, Salt Lake City, UT; bioMérieux, Salt Lake City, UT; bioMérieux, Salt Lake City, UT; bioMérieux, Salt Lake City, UT; bioMérieux, Salt Lake City, UT; bioMérieux, Salt Lake City, UT; bioMérieux, Salt Lake City, UT; bioMérieux, Salt Lake City, UT

## Abstract

**Background:**

Untreated sexually transmitted infections adversely impact sexual health and reproductive outcomes. bioMérieux is developing the BIOFIRE® SPOTFIRE® Sexually Transmitted Infection (STI) Panel, a molecular test designed for a point-of-care setting to detect *Chlamydia trachomatis, Mycoplasma genitalium, Neisseria gonorrhoeae,* and *Trichomonas vaginalis* from vaginal swabs (Vswabs) or penile urine collected in eNAT® medium. This study evaluated the limit of detection (LoD) and inclusivity of the pathogen assays in a RUO panel prototype.

**Table 1.**
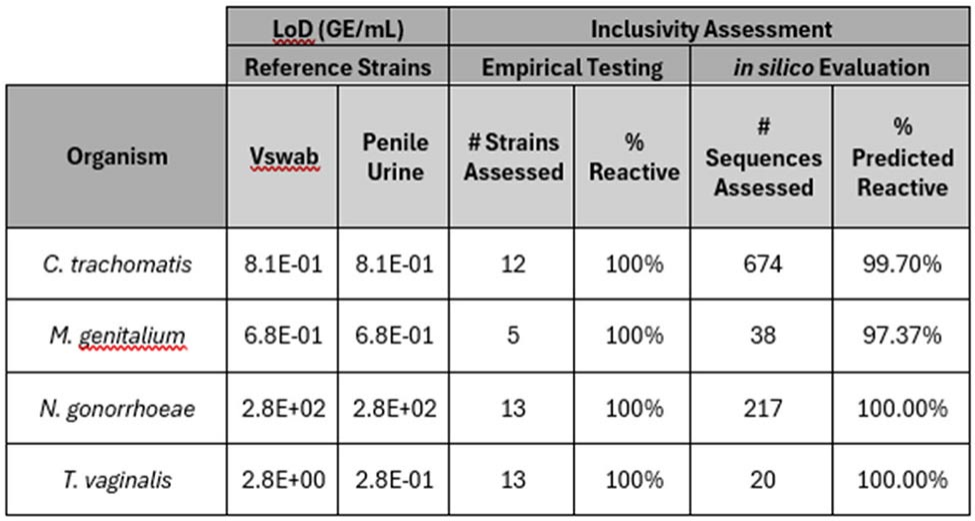
Limit of Detection and Inclusivity Assessment of the RUO BIOFIRE® SPOTFIRE® Sexually Transmitted Infection Panel

**Methods:**

Serial dilutions of reference isolates, quantified to determine genome copies using droplet digital PCR assays targeting single-copy genes, were spiked into negative clinical matrices and used to determine the 95% LoD for each pathogen. Inclusivity was assessed empirically using 43 PCR-quantified strains spiked into negative matrices at near-LoD genome equivalent (GE) levels as well as *in silico* using sequence data available at the time of analysis.

**Results:**

See Table 1 for study results. The LoD of the reference isolates varied from 275 GE/mL for *N. gonorrhoeae* to 0.28 GE/mL for *T. vaginalis*. All analytes besides *T. vaginalis* had equivalent LoDs in both sample matrices, with 10-fold greater sensitivity for *T. vaginalis* in contrived urine samples. Inclusivity testing of 43 organism strains at 5-20X LoD showed no reactivity limitations with equivalent reactivity in both sample matrices. *In silico* assessments based on assay primer homology to > 900 available sequences predict > 99.7% reactivity for 3 /4 analytes on the panel; an assessment of the degree of reactivity impairment for 2.6% of *M. genitalium* sequences is planned.

**Conclusion:**

This study suggests the BIOFIRE® SPOTFIRE® STI Panel will be capable of detecting low levels and multiple strains of clinically relevant *C. trachomatis*, *M. genitalium*, *N. gonorrhoeae,* and *T. vaginalis* strains from appropriate specimens.

**Disclosures:**

All Authors: No reported disclosures

